# Silencing NFBD1/MDC1 enhances the radiosensitivity of human nasopharyngeal cancer CNE1 cells and results in tumor growth inhibition

**DOI:** 10.1038/cddis.2015.214

**Published:** 2015-08-06

**Authors:** Z Wang, Q Zeng, T Chen, K Liao, Y Bu, S Hong, G Hu

**Affiliations:** 1 Department of Otorhinolaryngology, The First Affiliated Hospital of Chongqing Medical University, Chongqing, China; 2 Department of Oncology, The First Affiliated Hospital of Chongqing Medical University, Chongqing, China; 3 Department of Biochemistry and Molecular Biology, Molecular Medicine and Cancer Research, China Center, Chongqing Medical University, Chongqing, China

## Abstract

NFBD1 functions in cell cycle checkpoint activation and DNA repair following ionizing radiation (IR). In this study, we defined the NFBD1 as a tractable molecular target to radiosensitize nasopharyngeal carcinoma (NPC) cells. Silencing NFBD1 using lentivirus-mediated shRNA-sensitized NPC cells to radiation in a dose-dependent manner, increasing apoptotic cell death, decreasing clonogenic survival and delaying DNA damage repair. Furthermore, downregulation of NFBD1 inhibited the amplification of the IR-induced DNA damage signal, and failed to accumulate and retain DNA damage-response proteins at the DNA damage sites, which leaded to defective checkpoint activation following DNA damage. We also implicated the involvement of NFBD1 in IR-induced Rad51 and DNA-dependent protein kinase catalytic subunit foci formation. Xenografts models in nude mice showed that silencing NFBD1 significantly enhanced the antitumor activity of IR, leading to tumor growth inhibition of the combination therapy. Our studies suggested that a combination of gene therapy and radiation therapy may be an effective strategy for human NPC treatment.

Nasopharyngeal carcinoma (NPC) is a non-lymphomatous, squamous cell carcinoma that occurs in the epithelial lining of the nasopharynx, which is a prevalent tumor in people of southern Chinese ancestry in southern China and Southeast Asia, and the incidence is still increasing.^1^ Although radiotherapy is routinely used to treat patients with NPC, local recurrences and distant metastasis often occur in 30–40% of NPC patients at advanced staged.^2^ Thus, new therapeutic strategies are required to improve the poor prognosis of NPC.

Among the various types of DNA damage, DNA double-strand breaks (DSBs) are the most serious and require elaborated networks of proteins to signal and repair the damage.^3^ It has recently been shown that the histone H2A variant H2AX specifically controls the recruitment of DNA repair proteins to the sites of DNA damage.^4^ H2AX is phosphorylated extensively on a conserved serine residue at its carboxyl terminus in chromatin regions bearing DSBs, which is mediated by members of the phos-phoinositide-3-kinase-related protein kinase (PIKK) family.^5, 6^ Of these PIKKs, ataxia telangiectasia mutated (ATM) and DNA-dependent protein kinase catalytic subunit (DNA-PKcs) phosphorylate H2AX in response to DSBs in a partially redundant manner.^7, 8^ NFBD1 (Nuclear Factor with BRCT Domain Protein 1), also known as MDC1 (mediator of DNA damage checkpoint protein 1), is a recently identified nuclear protein that regulates many aspects of the DNA damage-response pathway, such as intra-S phase checkpoint, G2/M checkpoint, spindle assembly checkpoint and foci formation of NBS/MRE/Rad50 (MRN complex), 53BP1 and BRCA1.^9, 10, 11, 12, 13^ Human NFBD1 comprises 2089 amino acid residues and has a predicted molecular weight of ∼220 kDa. Motifs found in the protein include an FHA (Forkhead Associated) domain, two BRCT (BRCA1 carboxy terminal) domains and around 20 in terminal repeats of ∼41 amino acid residues each.^14^ Following DNA damage, NFBD1 serves as a bridging molecule and directly interacts with ATM and phospho-H2AX (*γ*-H2AX) through its FHA and BRCT domains, respectively, which leads to the expansion of *γ*-H2AX region surrounding DNA strand breaks and provides docking sites for many DNA damage and repair proteins including the MRN complex, 53BP1, BRCA1, RNF8, RNF4 and so on, ensuring genomics stability.^11, 15, 16, 17, 18^ In mammalian cells, DSBs are mainly repaired by two mechanisms, homologous recombination (HR) or non-homologous end-joining (NHEJ).^19, 20, 21^ For NHEJ repair, it is estimated that following exposure to ionizing radiation (IR), 80–90% of the DSBs in G1 are rejoined with fast kinetics in a manner dependent upon the NHEJ core components, Ku, DNA-PKcs, XRCC4 and DNA ligase IV. In contrast, HR predominates in late S- and G2-phase cells, when the sister chromatid is available to act as the template, representing those normally repaired with slow kinetics, require Rad51, Rad52, Rad54, XRCC2, XRCC3, the Rad51 paralogs and the breast cancer susceptibility genes BRCA1 and BRCA2.^22, 23, 24, 25, 26^

Since NFBD1 contains protein–protein interaction domains, and participate in the DNA damage-response (DDR) pathway. However, the mechanism by which NFBD1 regulates so many aspects of the DNA damage-response pathway in NPC cells is not fully understood. In addition, the physiological function of NFBD1 in NPC cells has been not investigated. With these goals in mind, we generated NFBD1-knockdown NPC cells and studied the physiological function of NFBD1 in DDR.

## Results

### Lentivirus-mediated shRNA inhibited NFBD1 mRNA and protein expression in CNE1 cell lines

**Figure 1:**
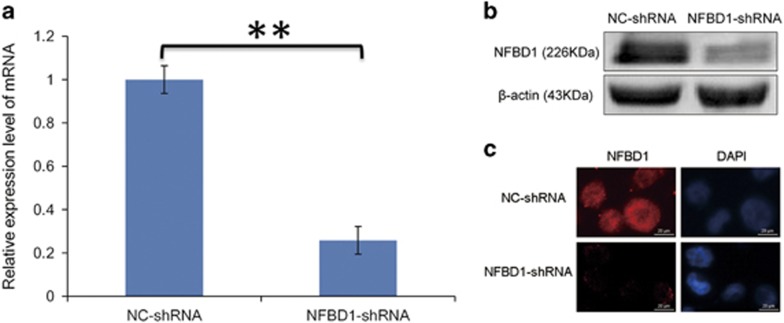
Knockdown of endogenous NFBD1 using lentivirus-mediated shRNA. The lentiviral expressing NFBD1 shRNA and control shRNA were transfected into CNE1 cells. The transfected cells of stable expression NFBD1 shRNA and NC-shRNA were obtain under puromycin (1 mg/ml). (**a**) Effects of constructed lentiviral on the expression of NFBD1 mRNA were determined by qRT-PCR. The relative expression level of NFBD1 mRNA was significantly downregulated in NFBD1-shRNA group, *P*<0.05 compared with NC-shRNA group. Effects of constructed lentiviral on the expression of NFBD1 protein were determined by western blotting (**b**) and immunofluorescence (**c**) *P<0.05 and **P<0.01

The lentiviral expressing NFBD1 shRNA and control shRNA were transfected into CNE1 cells. The transfected cells of stable expression NFBD1 shRNA and control shRNA were obtain under puromycin (1 *μ*g/ml).The expression of NFBD1 mRNA in NFBD1-shRNA group were decreased by (74.81±1.34)% compared with NC-shRNA group by real-time quantitative RT-PCR (qRT-PCR) (Figure 1a). The NFBD1 protein in NFBD1-shRNA group compared with NC-shRNA group was significantly decreased by western blotting (Figure 1b). Immunofluorescence also revealed corresponding decrease in the protein levels (Figure 1c). These results demonstrated that the lentivirus-mediated shRNA targeting NFBD1 effectively ‘knocked down' NFBD1 expression at both mRNA and protein levels in the CNE1 cells.

### Silencing NFBD1 moderately sensitizes CNE1 cells to ionizing radiation

**Figure 2:**
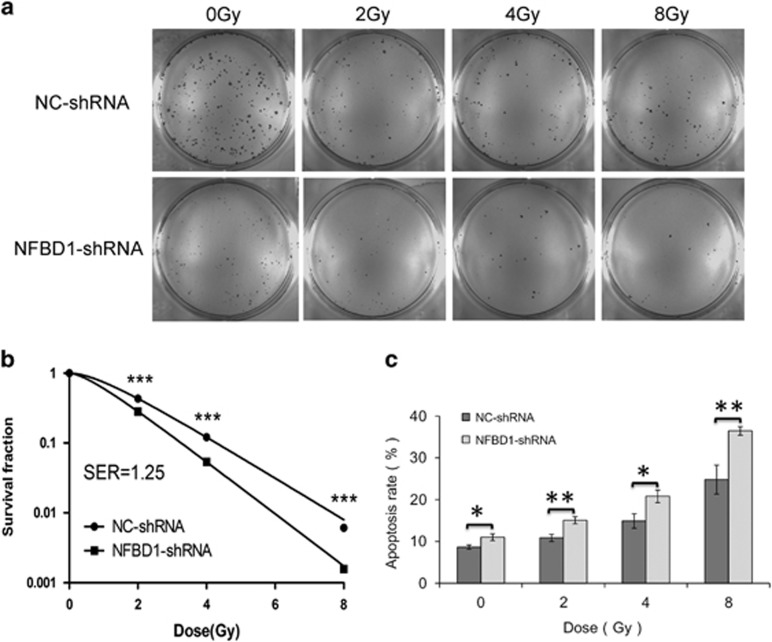
Knockdown of NFBD1 expression affects radiosensitivity. (**a** and **b**) Cells were plated at low density, irradiated and colonies counted after 14 days. Results are normalized for effects of NFBD1 and fitted to a standard linear quadratic model. (**c**) The percentage of apoptotic cells (early apoptosis+late apoptosis) was measured using Annexin V and propidium iodide with flow cytometry. *P<0.05, **P<0.01 and ***P<0.001

We next examined the ability of NFBD1 silenced cells to respond to IR-induced DNA damage, cells were plated at low density, exposed to IR and assessed for their ability to form colonies. NFBD1-shRNA group exhibited hypersensitivity to the killing effects of IR (Figures 2a and b) when compared with the NC-shRNA group. Furthermore, NFBD1-shRNA group also reduced the number of colonies formed from undamaged cells when compared with NC-shRNA group, and the sensitizing enhancement ratio (SER) was 1.25 (Figure 2b), indicating that NFBD1 function may be required to maintain cell viability. Considering the notion that cells can undergo apoptosis when DNA damage is irreparable, therefore, to examine whether downregulation of NFBD1 following radiation can induce apoptosis, NFBD1-shRNA group and NC-shRNA group cells were radiated at 2, 4 and 8 Gy, respectively, and determined the apoptosis rate at 48 post IR using flow cytometry (Figure 2c). IR potently induced apoptosis in CNE1 cells in a dose-dependent manner; furthermore, NFBD1 knockdown significantly enhanced radiation-induced apoptosis. These results indicated that downregulation of NFBD1 can enhance NPC cell CNE1 radiosensitivity.

### NFBD1 participates in the regulation of G2/M phase checkpoint and IR-induced DNA damage repair

**Figure 3:**
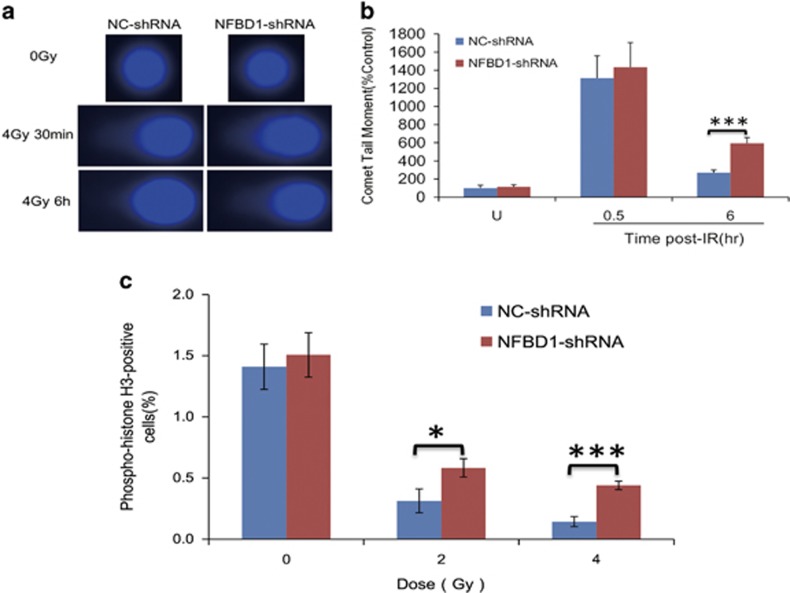
Analysis of the G2/M checkpoint and DNA damage repair. (**a** and **b**) The repair of DNA damage was detected by comet assay. Representative images were on the left. (**a**) The comet tail moment of 75 cells for each time and condition was quantified by CASP software and normalized to that of no irradiation. (**b**) The comet tail moment of each time and condition was quantified by CASP software and normalized to that of no irradiation. (**b** and **c**) Cells were untreated or irradiated as indicated, then incubated for 1 h at 37 °C before fixation. Mitotic cells were determined by phospho-histone H3 staining and flow cytometry. Each analysis was performed by using 20 000 cells. *P<0.05 and ***P<0.001

DNA DSBs are the most dangerous damage caused by IR, posing a serious threat to cell viability and genome stability. Considering the notion that apoptosis can increase DNA damage and histone H3 phosphorylation, so we determined if IR induces the apoptosis in the early phase, the results showed that the apoptotic percentage was not significantly unchanged post IR treatment (Supplementary Figure S1). To determine whether NFBD1 knockdown affects DNA repair in the early phase post IR, we measured the persistence of DSB in IR-treated CNE1 cells utilizing single-cell gel electrophoresis (comet assay, Figures 2a and b). IR treatment induced DSBs, visibly by increased DNA mobility or ‘comet tail'. 30 min after IR treatment, NFBD1-shRNA group and NC-shRNA group cells had comparable amounts of DNA damage. However, DNA repair was essentially completed by 6 h in the control population, whereas tails were still visible in NFBD1-shRNA group before IR-inducing apoptosis was occurred. Thus, NFBD1 was required for IR-induced DNA damage repair. Besides triggering apoptosis, DNA damage also induces an evolutionarily conserved DNA damage-response to activate cell cycle checkpoints to arrest cell cycle progression and allow for DNA repair so as to preserve genomic integrity.^27, 28^ Therefore, to examine whether NFBD1 participates in the regulation of G2/M phase checkpoint after IR, cells were irradiated with 2 and 4 Gy IR, respectively, and the G2/M checkpoint function was determined by monitoring histone H3 phosphorylation on serine 10.^29, 30^ Clear reduction in phospho-H3-positive cells was observed in the NC-shRNA group cells at 1 post IR exposure, whereas a significant number of the cells lacking NFBD1 entered mitosis in the absence of IR-induced apoptosis induction (Figure 3c; Supplementary Figure S1), indicative of a defect in the ability to arrest the cell cycle in G2 phase.

### NFBD1 is required for IR-induced p-ATM and *γ*-H2AX foci formation

**Figure 4:**
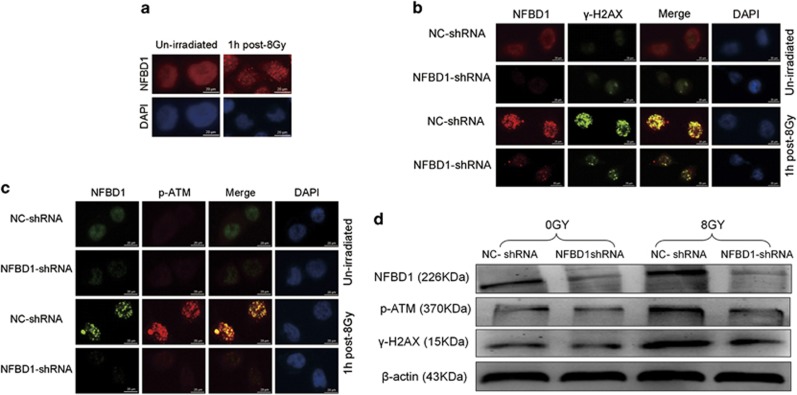
NFBD1 regulates *γ*-H2AX and p-ATM foci formation. (**a**) IR-induced NFBD1 foci formation. Cells were untreated or irradiated with 8 Gy, fixed and stained with anti-NFBD1 antibodies at the times indicated. (**b**) and (**c**) Inhibition of NFBD1 results in defective p-ATM and *γ*-H2AX foci formation after IR exposure. Cells were irradiated with 8 Gy IR, fixed at 1 post irradiation and stained with the indicated antibodies (**b**: mouse anti-ATM (pT1981) and rabbit anti-NFBD1; **c**: rabbit anti-*γ*-H2AX and mouse anti-NFBD1). (**d**) CNE1 cells were untreated or irradiated with 8 Gy IR, harvested 1 h later and subjected to western blotting analysis with indicated antibodies. Representative blots were shown with *β*-actin as loading control

To assess whether NFBD1 could localize to sites of damage, cells were treated with IR and stained with anti-NFBD1 antibody. NFBD1 rapidly formed foci within 1 h after exposure to IR (Figure 4a). A proportion of unirradiated cells also contained NFBD1 foci, indicating that NFBD1 may be responding to endogenous damage or replication stress. ATM activation is critical for response to DNA damage characterized by DSBs.^15, 31^ In mammalian cells, ATM is recruited into IR-induced DSB sites, and phosphorylation H2AX (*γ*-H2AX) may highlight the damaged chromatin.^32^ As NFBD1 controls the phosphorylation of several checkpoint-responsive proteins, we sought to examine whether it might have a role in ATM and H2AX phosphorylation. Silencing NFBD1 significantly affected the phosphorylation of H2AX and ATM after IR and the formation of *γ*-H2AX and p-ATM foci, the same result was observed in western blotting analysis (Figure 4d). Furthermore, NFBD1 foci also significantly co-localized with *γ*-H2AX and p-ATM foci at 1 post irradiation (Figures 4b and c). Thus NFBD1 was necessary for ATM and H2AX phosphorylation and foci formation after DNA damage.

### Down-regulation NFBD1 results in impaired Rad51 and DNA-PKcs foci formation

**Figure 5:**
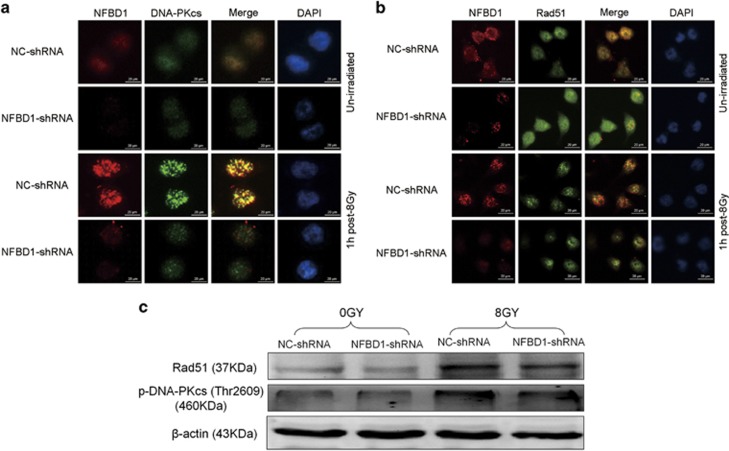
NFBD1 regulates DNA-PKcs and Rad51 foci formation. (**a** and **b**) Inhibition of NFBD1 resulted in defective DNA-PKcs and Rad51 foci formation after IR exposure. Cells were untreated or irradiated with 8 Gy IR, fixed at 1 h post irradiation and stained with the indicated antibodies (mouse anti-NFBD1, rabbit anti-DNA-PKcs (pT2609) and rabbit anti-Rad51). (**c**) CNE1 cells were untreated or irradiated with 8 Gy IR, harvested 1 h later and subjected to western blotting analysis with indicated antibodies. Representative blots were shown with *β*-actin as loading control

The Rad51 and phosphorylated DNA-PKcs proteins are frequently detected in multiple discrete subnuclear structures, referred to as nuclear foci, which are thought to participate in the DNA repair process and may represent sites of ongoing recombination. To define the relationship between NFBD1 and Rad51, phosphorylated DNA-PKcs, we studied the formation of Rad51 IR-induced foci (IRIF) after 8 Gy IR. The results of immunofluorescence staining displayed that downregulation NFBD1 significantly affected the formation of Rad51 and DNA-PKcs foci after 8 Gy IR (Figures 5a and b). The same result was observed in western blotting analysis (Figure 5c). Furthermore, NFBD1 foci also significantly co-localized with DNA-PKcs foci at 1 post irradiation (Figure 5a), but not co-localized with Rad51 (Figure 5b). Thus it can be seen that both Rad 51 and phosphorylated DNA-PKcs were impaired after NFBD1 knockdown following IR.

### NFBD1 maintains Rad51 and DNA-PKcs in chromatin and the nucleus

**Figure 6:**
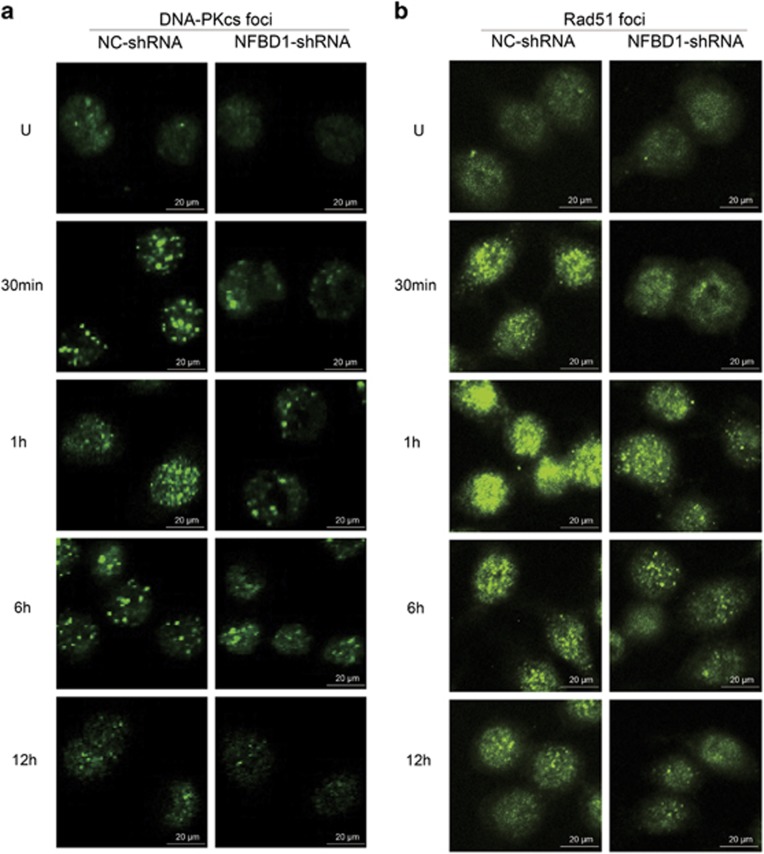
Representative Rad51 and DNA-PKcs foci in cells NC-shRNA or NFBD1-shRNA. Rad51 and DNA-PKcs foci formation were deficient in NFBD1-knockdown cells at early time points after exposure to ionizing radiation. Cells were exposed to 8 Gy and fixed for immunocytochemical analysis of Rad51 and DNA-PKcs at the indicated times. Subnuclear aggregates of Rad51 and DNA-PKcs (foci) were visualized by immunofluorescence staining

As previously reported, upon IR agents, the Rad51 and DNA-PKcs proteins were recruited to the end of resulting DNA damage and formed subnuclear complexes that were microscopically detectable as foci, which contained many of the enzymatic activities required for efficient repair of DSBs. To further identify the relationship of NFBD1 with the two critical proteins, we studied the formation of Rad51 and DNA-PKcs IRIF at various time points after exposure to 8 Gy of IR (Figures 6a and b). The formation of Rad51 and DNA-PKcs foci at early time points (within 1 h) was significantly different between NC-shRNA and NFBD1-shRNA groups. In addition, the Rad51 and DNA-PKcs foci-positive cells transfected with control shRNA contained both more and brighter foci than the cells transfected with NFBD1-shRNA, especially at early time points. These results indicated that NFBD1 maintained Rad51 and DNA-PKcs in chromatin and the nucleus at early time after IR.

### Silencing NFBD1 enhances CNE1 cells' response to radiation and results in tumor growth inhibition *in vivo*

**Figure 7:**
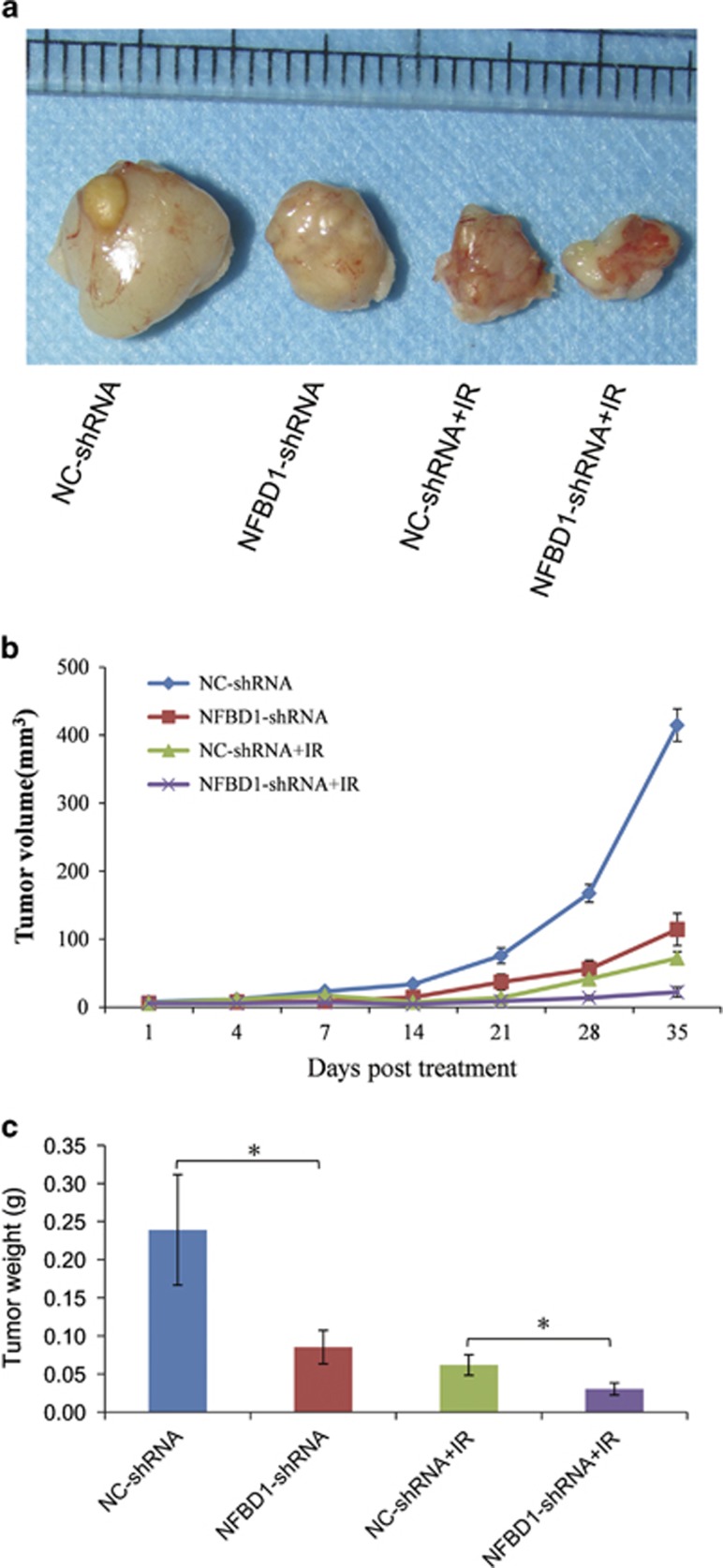
Combination therapy inhibits tumor growth of nasopharyngeal cancer *in vivo*. Radiation (2 Gy) was delivered to tumors at day 3, 5, 7 and 9 using a linear accelerator in radiation group. The tumors were then measured twice a week using a digital caliper. Tumor volume=1/2 × length × width^2^. (**a**) Representative images of the tumors from mice 35 days after treatments. (**b**) The growth curves of CNE1 NC-shRNA and NFBD1-shRNA xenografts with radiation or non-radiation treatment. (**c**) Tumor weight at 35 days after treatments, *n*=5 mice per condition. *P<0.05. IR: ionizing radiation

To determine the effect of combining downregulation NFBD1 with radiation *in vivo*, we next set up xenograft model in athymic nude mice. We measured tumor volume twice per week to determine whether silencing NFBD1-enhanced CNE1 cells' response to radiation. Our results showed that either downregulation of NFBD1 or irradiation alone resulted in significantly smaller tumor than untreated xenografts. However, the combination of downregulated NFBD1 and irradiation resulted in significantly smaller tumors as compared with untreated controls or to tumors treated with downregulated NFBD1 or irradiation alone (Figures 7a–c). Thus, the results suggested that silencing NFBD1 enhanced the response of nasopharyngeal cancer CNE1 cells to radiation and resulted in tumor growth inhibition *in vivo*.

## Discussion

Radiation therapy is used to treat all stages of localized NPC. Although it is very effective in local control, local recurrences and distant metastasis often occur in 30–40% of NPC patients at advanced staged,^2^ and majority of patients will also ultimately die of their disease, suggesting that further studies are needed to develop new strategies to improve radiation-dependent tumor cytotoxicity and improve the prognosis of patients with NPC. Gene therapy is a therapeutic option that holds great promise for the treatment of monogenic diseases as well as various forms of cancer. To June 2012 over 1800 gene therapy clinical trials have been completed, are ongoing or have been approved worldwide.^33^ Engineered viruses are often exploited to efficiently deliver therapeutic genes to the target cells. The choice of the viral vector to be used in these protocols is based on a careful evaluation of vector design and transduction properties, manufacturing capacity and safety of the platform. HIV-derived lentiviral vectors display features that render them particularly useful for gene therapy.^34^

NFBD1 has important roles in DNA damage response (DDR), involving a complex network of signaling pathways that regulates cell cycle checkpoints, DNA repair and cell death.^35, 36, 37, 38^ To understand the role of NFBD1 in NPC, a stable NPC cell line NFBD1-shRNA was first developed, which expressed downregulation of NFBD1. In the study, we found that silencing NFBD1 by lentivirus-mediated shRNA significantly enhanced radiation-induced apoptosis and growth inhibition of human NPC CNE1 cells. More importantly, NFBD1 knockdown can delay IR-induced DNA damage repair in the early phase of the DDR, also this enhanced radiosensitivity was accompanied by an increased induction of apoptosis in a later phase, inhibited growth, xenografts models in nude mice showed that silencing NFBD1 significantly enhanced the antitumor activity of IR, leading to tumor growth inhibition of the combination therapy. These findings suggested that NFBD1 may be potential therapeutic targets to increase radiosensitivity of human NPC.

The sensitivity to IR suggests an important role in responding to DNA damage. It has been argued that cell cycle checkpoint arrest, although important for maintaining genomic stability post irradiation, makes a less significant contribution to survival. However, when cell cycle checkpoint defects are combined with defective DSB repair, the impact is more than additive, consistent with the notion that cell cycle checkpoint arrest enhances the opportunity for DSB repair.^39^ In our studies, we found that a clear reduction in M phase cells was observed in the control-treated cells in the early phase after IR exposure, whereas a significant number of the cells lacking NFBD1 entered mitosis, indicative of a defect in the ability to arrest the cell cycle in G2 phase. Furthermore, we also found that downregulation NFBD1 impaired the formation of Rad51 and DNA-PKcs IRIF which were respectively known as the representative proteins of HR and NHEJ-mediated repair pathways. From these data, silencing NFBD1-enhanced radiosensitivity of CNE1 cells through impairing G2/M checkpoint activity and DNA damage repair in the early phase post IR.

In the case of IR-induced DSBs, *γ*-H2AX is ATM-dependent, which is involved in the amplification step required for optimal checkpoint response in the DDR.^4, 40^ It is evident that a network of interactions is initiated around *γ*-H2AX, which recruits and maintains many DDR proteins at sites of DSBs.^41, 42, 43, 44, 45^ Within 1 h after IR, NFBD1 was found in nuclear foci, which also seemed to contain *γ*-H2AX and p-ATM (pT1981), the two proteins known to be involved in early DDR, and knockdown of NFBD1 greatly reduced IR-induced formation of *γ*-H2AX and p-ATM foci, The same result was observed in western blotting analysis. Moreover, our co-localization studies indicated NFBD1 foci extensively overlap with *γ*-H2AX and p-ATM (pT1981) foci, suggesting that NFBD1 served as a bridging molecule between *γ*-H2AX and p-ATM. These results indicated that downregulation of NFBD1 can inhibited the amplification of the IR-induced DNA damage signal, and failed to accumulate and retain DDR proteins at the sites of DNA damage, which leaded to defective checkpoint activation following DNA damage.

As far as we know, the phosphorylation of NHEJ components, including DNA-PKcs itself, is important for the intrinsic kinase activity of DNA-PK, which accounts for NHEJ-mediated DSB repair and the cellular resistance to radiation, however, Rad51 protein, has an essential role exclusively in HR in mammalian cells.^26, 46, 47, 48^ Our data both by western blotting and nuclear foci formation, confirmed that depleting cells of NFBD1 protein significantly affected the formation of Rad51 and DNA-PKcs foci upon irradiation, especially at early time points after DNA damage. Furthermore, NFBD1 foci also significantly co-localized with DNA-PKcs foci at 1 post irradiation, but not co-localized with Rad51. Thus we speculated that NFBD1 may affect DNA repair via the NHEJ pathway through its direct interaction with DNA-PK, while the main mechanism of NFBD1 in HR may be mediated by the regulation of Rad51 stability, which affected its recruitment to DSBs and subsequent repair. Furthermore, Lou *et al.*^15^ found that NFBD1^−/−^ mice displayed growth retardation, male infertility, immune defects, chromosome instability, DNA repair defects and radiation sensitivity. In the current study, our data showed that downregulation of NFBD1 significantly improved antitumor activity of IR to CNE1 cells *in vivo,* resulting in tumor regression. Given the fact that many of these checkpoint proteins are well-characterized cancer targets and many of their inhibitors are currently being developed at the different phases of clinical trials,^49, 50^ therefore, the finding that silencing NFBD1 impairs G2/M checkpoint and DNA damage repair makes NFBD1 a more appealing anticancer target. But up to now, we have not found NFBD1 related inhibitors, thus the development of NFBD1 inhibitors would be urgently needed in future study.

In summary, we report here that NFBD1 knockdown by lentivirus-mediated shRNA can inhibit cell growth, alter cell cycle progression, induce apoptosis and moderately sensitize CNE1 cells to radiation. NFBD1 knockdown also inhibit the amplification of the IR-induced DNA damage signal, and fail to accumulation and retain DDR proteins at the sites of DNA damage, which leads to defective checkpoint activation following DNA damage. Furthermore, silencing of NFBD1 impairs the formation of Rad51 and DNA-PKcs IRIF. Using xenografts models in nude mice showed that silencing NFBD1 significantly enhanced the antitumor activity of IR, leading to tumor growth inhibition of the combination therapy. Our studies provide compelling evidence that combining depletion of NFBD1 and radiation represents a rational strategy for the treatment of patients with NPC.

## Materials and methods

### Cell culture

CNE1, an EBV-negative human NPC cell line, was obtained from the Molecular Medicine and Cancer Research Center, Chongqing Medical University. The cells were grown in RMPI-1640 medium (HyClone, Logan City, Utah, USA) with 10% fetal bovine serum (HyClone, Logan City, Utah, USA) at 37 °C with 5% CO_2_.

### Lentivirus-mediated shRNA downregulation of gene expression

The shRNA oligonucleotide or a lentivirus-mediated shRNA (Genechem, Shanghai, China) construct was used to silence NFBD1. The sequence of shRNA oligonucleotide for positive experiment group (NFBD1-shRNA) 5′-GAGGCAGACUGUGGAUAAATT-3′, Nonsilencing sequence 5′-TTCTCCGAACGTGTCACGT-3′ was used as a control, which was named negative control group (NC-shRNA).

The CNE1 cells were transferred into six-well plates by the density of 2 × 10^4^/well and divided into two groups, NFBD1-shRNA group and NC-shRNA group. Cells were cultured in 1 ml medium (contain 4 *μ*g/*μ*l polybrene) and 2 *μ*l viral supernatants (the number of viruses are 2 × 10^5^). 24 h later, culture medium was replaced with normal medium. After 72 h, the cells were cultured in the mediun with 1* μ*g/ml puromycin to select cells that have been transduced with the lentivirus containing NFBD1-shRNA or NC-shRNA. Seven days later, the alive cells were collected and cultured for cell amplification. The qRT-PCR, western blotting and immunofluorescence were used to detect the inhibition rate of lentivirus-mediated shRNA targeting NFBD1.

### Real-time quantitative RT-PCR (qRT-PCR)

Total RNA was extracted by using Trizol (Invitrogen, Carlsbad, CA, USA) according to the manufacturer's instructions. Total RNA (500 ng) was reverse transcribed into cDNA using One-Step SYBR PrimeScript RT-PCR Kit II (Takara Biotechnology, Dalian, China) by the supplied protocol. The qRT-PCR reaction was performed using the SYBR Premix Ex Taq (Takara Biotechnology) in a LightCycler 480 qRT-PCR system (Bio-RAD CFX96, Hercules, CA, USA) according to the manufacturer's instructions. The primers for NFBD1 were 5′-AGCAACCCCAGTTGTCATTC-3′ (forward), 5′-TCAGATGTGCCAAAGTCAGC-3′ (reverse), GAPDH was used as the internal standard in *qRT-PCR* system. The primers for GAPDH were 5′-ACCTGACCTGCCGTCTAGAA-3′ (forward), 5′-TCCACCACCCTGTTGCTGTA-3′ (reverse). The results were analyzed by the ΔΔCt method. Each experiment was repeated three times.

### Antibodies and western blotting

The antibodies used in this study were: mouse and rabbit anti-NFBD1 (Abcam, London, UK); anti-ATM (phospho S1981) (Abcam); anti-DNA PKcs (phospho T2609) (Abcam); anti-phospho-H2AX (Ser139) (Cell Signaling Technology, Danvers, MA, USA); anti-phospho-histone H3 (Ser10) (Cell Signalling Technology); rabbit anti-Rad51 (Santa Cruz Biotechnology, Dallas, TX, USA). Total protein extracts from cells were prepared using RIPA buffer (Beyotime Institute of Biotechnology, Nantong, China). Protein concentrations were determined using the BCA protein assay systems (Beyotime Institute of Biotechnology, Nantong, China). Proteins were fractionated in SDS–polyacrylamide gels. Proteins were transferred to polyvinylidene fluoride (Millipore, Billerica, MA, USA), and western blotting were performed by using the appropriate antibody. Antibody/antigen complexes were detected using ECL (Western Bright Sirius; Advansta, Inc., Menlo Park, CA, USA) and images were acquired using an enhanced chemifluorescence detection system (Amersham Biosciences, Piscataway, NJ, USA) under the room temperature.

### Immunofluorescence

Cells were fixed with 4% paraformaldehyde at room temperature for 30 min, permeablized with 0.5% Triton X-100 solution, washed three times in PBS for 5 min, blocked using 5% fetal bovine serum in PBS, and then primary antibodies were applied overnight at 4 °C, and secondary antibodies (green, Invitrogen; red, Beyotime Institute of Biotechnology, Nantong, China) were applied for 60 min at room temperature. Finally, the cells were washed three times in PBS, and the DNA was stained using DAPI (Sigma-Aldrich, St. Louis, MO, USA) at 50ng/ml. The slides were observed under a Nikon Microphot-FX fluorescence microscope (Tokyo, Japan).

### Colony-forming assay

Cells were seeded at low density and irradiated with various doses of IR. Cells were left for 14 days at 37 °C with 5% CO_2_ to allow the colonies to form. Cells were fixed with 70% ethanol for 20 min and then stained with 0.5% crystal violet for 20 min. Colonies containing 50 or more cells were counted as survivors.

### Apoptosis assays

CNE1 cells were seeded onto six-well plates at the density of 10 × 10^4^ cells per well and irradiated with various doses of IR. The cells were then incubated for 48 h, and stained with FITC-conjugated Annexin V and propidium iodide (PI), using Annexin V-FITC Apoptosis Detection kit (Beyotime Institute of Biotechnology, Nantong, China) and according to manufacturer's recommendation. Each sample was then subjected to analyses by flow cytometry using a FACSVantage SE system (BD Biosciences, San Jose, CA, USA). The percentage of apoptotic cells was determined by Flow cytometry.

### G2/M checkpoint recovery assay

CNE1 cells were seeded onto six-well plates at the density of 10 × 10^4^ cells per well, irradiated with various doses of IR as indicated, and then incubated for 1 h at 37 °C. The cells were fixed with ethanol, re-suspended in PBS containing 0.25% (vol/vol) Triton X-100, incubated on ice for 15 min and then incubated in PBS containning 1% BSA and phospho-histone H3 (Ser10) antibody for 1 h at room temperature. Samples were then incubated for 30 min at room temperature with Alexa fluor 488 donkey anti-rabbit conjugated secondary antibodies and were determined analyzed by a FACSVantage SE system (BD Biosciences) and analyzed by using CELLQUEST software. Each analysis was performed by using 20 000 cells.

### Neutral single-cell gel electrophoresis assay (Comet assay)

The Comet Assay kit (Trevigen Inc., Gaithersburg, MD, USA) was used under neutral conditions according to the manufacturer's specifications. Cells were treated with 4 Gy or sham treated and collected at 0 h (no-IR), 30 min and 6-post IR. Cells were re-suspended in 10% low-melting-point agarose and immediately pipette 50 *μ*l onto two-well comet assay slides. Once the agarose solidified, the slides were added to a bath of cell lysis solution (Trevigen Inc) overnight at 4 °C. The following day, the samples (i.e., the free DNA fragments) on the slides were electrophoresed and stained using DAPI (Sigma-Aldrich). Comets were visualized using a Nikon Microphot-FX fluorescence microscope. The tail moments (TMs) of comets were scored using CASP software.

### *In vivo* tumourigenicity

All animal husbandry and experiments were performed under a protocol approved by Institutional Animal Care Committee at Chongqing Medical University. CNE1 NC-shRNA cells (6.0 × 10^6^) were subcutaneously injected into the left gluteal region of 10 nude mice, and CNE1 NFBD1-shRNA cells (6.0 × 10^6^) were subcutaneously injected into the right gluteal region of the same ten nude mice. Then the ten treated nude mice were randomized into radiation group and non-radiation group. Each group was consisted of five mice. The radiation group was received 2 Gy of radiation at day 3, 5, 7 and 9 using a linear accelerator (Clinac 2300C/D; Varian, Palo Alto, CA, USA). The tumors were then measured twice a week using a digital caliper. Tumor volume=1/2 × length × width^2^. Mice were killed after 35 days after treatment, and then the tumors in the left and right gluteal region were excised and weighed.

### Statistics

Statistical comparison of mean values was performed using ANOVA, rank sum test (non-parametric statistics) or chi-square (*χ*^2^) test. Differences with a *P*-value of <0.05 were considered statistically significant.

## Abbreviations

IR: ionizing radiation
NPC: nasopharyngeal carcinoma
HR: homologous recombination
NHEJ: non-homologous end-joining
DSBs: DNA double-strand breaks
PIKK: phos-phoinositide-3-kinase-related protein kinase
ATM: ataxia telangiectasia mutated
DNA-PKcs: DNA-dependent protein kinase catalytic subunit
NFBD1: Nuclear Factor with BRCT Domain Protein 1
MDC1: Mediator of DNA damage checkpoint protein 1
MRN: MRE1/1NBS1/RAD50
SER: sensitizing enhancement ratio
IRIF: ionizing radiation-induced foci
qRT-PCR: real-time quantitative RT-PCR
Gy: Gray
TMs: tail moments
DDR: DNA damage response
