# Supplementary material for: Silencing NFBD1/MDC1 enhances the radiosensitivity of human nasopharyngeal cancer CNE1 cells and results in tumor growth inhibition
Source: Cell Death Dis. 2015 Aug 6;6(8):e1849–. doi: 10.1038/cddis.2015.214 (PMC4558506; doi:10.1038/cddis.2015.214)
Supplement: Supplementary Figure Legend [file cddis2015214x1.doc]

**Supplemental Figure Legends**

**Supplemental Figure S1.** Apoptotic response at 1h and 6h post-4Gy treatment. The percentage of apoptotic cells (early apoptosis+late apoptosis) was measured using Annexin V and propidium iodide with flow cytometry. Each bar represents the mean ± SD of three independent experiments (n = 3). U^＃^: un-irradiation.
