# Supplementary figures and images for: Silencing NFBD1/MDC1 enhances the radiosensitivity of human nasopharyngeal cancer CNE1 cells and results in tumor growth inhibition
Source: Cell Death Dis. 2015 Aug 6;6(8):e1849–. doi: 10.1038/cddis.2015.214 (PMC4558506; doi:10.1038/cddis.2015.214)

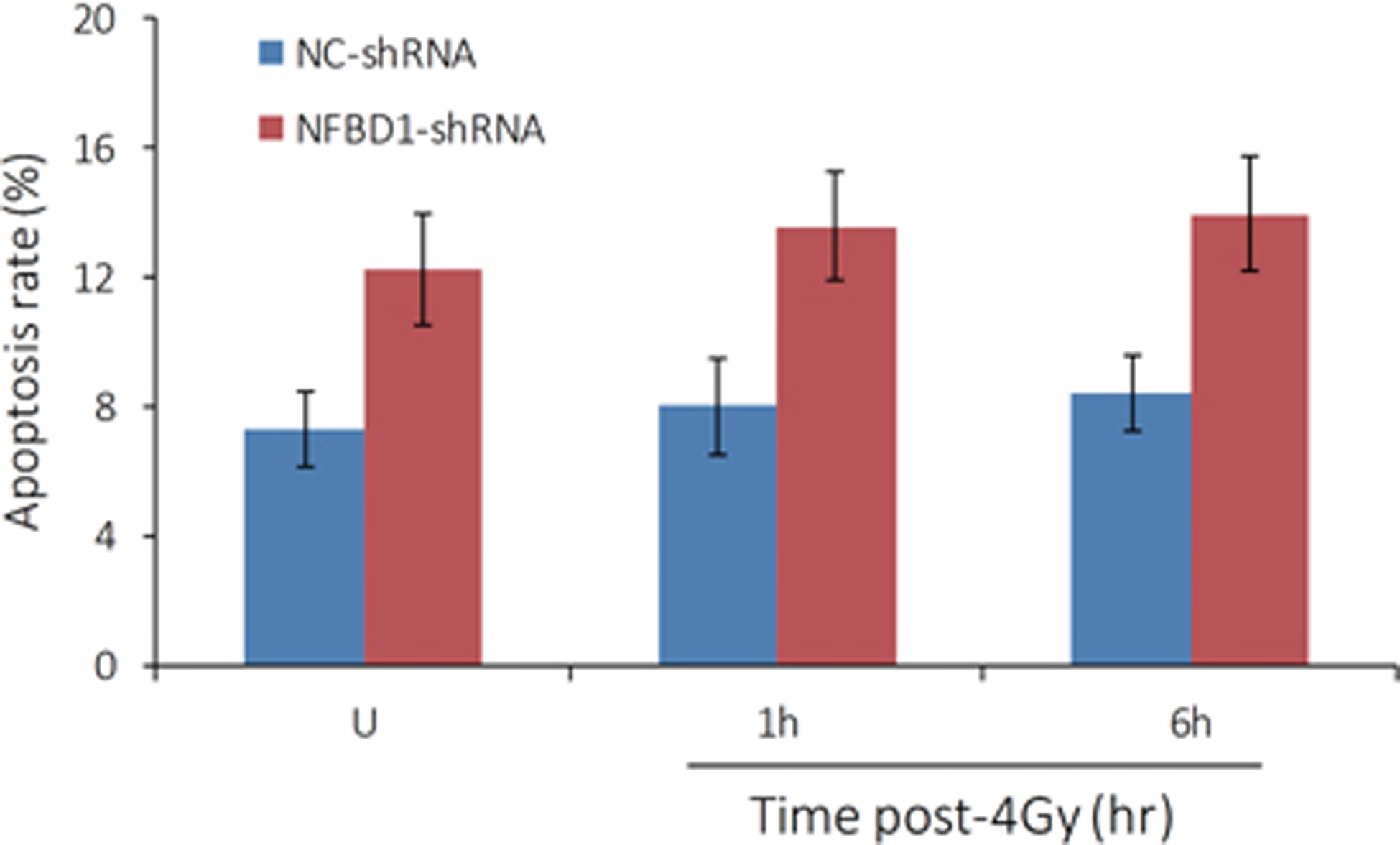

Supplement: Supplementary Figure S1 [file cddis2015214x2.tif]
